# ‘Being in a womb’ or ‘playing musical chairs’: the impact of place and space on infant feeding in NICUs

**DOI:** 10.1186/1471-2393-13-179

**Published:** 2013-09-23

**Authors:** Renée Flacking, Fiona Dykes

**Affiliations:** 1 Maternal and Infant Nutrition and Nurture Unit (MAINN), School of Health, University of Central Lancashire, Preston, Lancashire, UK; 2 School of Health and Social Studies, Dalarna University, Falun, Sweden

**Keywords:** Breastfeeding, Ethnography, Geography, Infant feeding, Neonatal, NICU, Place, Space

## Abstract

**Background:**

Becoming a parent of a preterm baby requiring neonatal care constitutes an extraordinary life situation in which parenting begins and evolves in a medical and unfamiliar setting. Although there is increasing emphasis within maternity and neonatal care on the influence of place and space upon the experiences of staff and service users, there is a lack of research on how space and place influence relationships and care in the neonatal environment. The aim of this study was to explore, in-depth, the impact of place and space on parents’ experiences and practices related to feeding their preterm babies in Neonatal Intensive Care Units (NICUs) in Sweden and England.

**Methods:**

An ethnographic approach was utilised in two NICUs in Sweden and two comparable units in England, UK. Over an eleven month period, a total of 52 mothers, 19 fathers and 102 staff were observed and interviewed. A grounded theory approach was utilised throughout data collection and analysis.

**Results:**

The core category of ‘the room as a conveyance for an attuned feeding’ was underpinned by four categories: the level of ‘ownership’ of space and place; the feeling of ‘at-homeness’; the experience of ‘the door or a shield’ against people entering, for privacy, for enabling a focus within, and for regulating socialising and the; ‘window of opportunity’. Findings showed that the construction and design of space and place was strongly influential on the developing parent-infant relationship and for experiencing a sense of connectedness and a shared awareness with the baby during feeding, an attuned feeding.

**Conclusions:**

If our proposed model is valid, it is vital that these findings are considered when developing or reconfiguring NICUs so that account is taken of the influences of spatiality upon parent’s experiences. Even without redesign there are measures that may be taken to make a positive difference for parents and their preterm babies.

## Background

There has been a growing interest in the ways in which configuration and construction of place and space within hospitals influence health, health behaviours and relationships [1]. The concepts of place and space may be defined as geometric constructs; “space as a container and place as locations” [2], p.716. However, place is not merely a setting but has evident and independent effects on social life [3] in which architectures of, for example enclosure, display or surveillance contribute to estrangements and/or submissive behaviours [3,4]. Meanings that individuals assign to a place will be embedded in a shared cultural understanding of that environment [3], and although space can be defined as ‘a container’ , which is filled with people, practices, objects, values and representations [2,3], the notion of space includes social constructions where space is a “[..] medium through which the character of places are reproduced [..]” [2], p.715.

Within maternity and neonatal care, the influence of place and space upon the experiences of staff and service users has received an increased focus [5,6]. This follows from a well-rehearsed critique that relates to the medicalization and institutionalisation of maternity care that took place over the twentieth century resulting in a change of place of birth from home to hospital and associated dehumanising environments and practices [7-12]. As a result, there are moves to humanise the environments of care, for example the birth centre movement endeavours to provide a place like ‘home’ within a small maternity unit setting [11,12]. Another area which is being addressed in the actual design of physical places is in neonatal intensive care units (NICUs) [5]; in particular, there is a trend in some countries towards single family room design when building new units [5,6] increasingly replacing traditional open-bay design units. This design provides the family with an opportunity to be with their baby, in the NICU, day and night, in privacy [13]. Consequently, this design has been associated with earlier full enteral nutrition, higher breastfeeding rates, a more soothing environment and shorter hospital stay which, in turn, shortens the time of separation for the baby from the home and family [14,15]. Even in traditional open bay units there are ways to facilitate opportunities for parents to be close to the baby, for example by providing comfortable chairs and/or beds for them. However, there is a very large variation between NICUs as to the extent to which they offer such facilities [16].

The birth of a preterm baby places the mother-baby relationship under pressure, as the maternal role and feeding begin and develop within a highly medicalised and unfamiliar setting [17]. Breast milk is of vital importance for nutritional, immunological, and cognitive development [18] and these advantages are even more pronounced in preterm babies [19,20]. Hence, much effort is made in NICUs to facilitate the initiation and duration of breast milk provision to babies. Studies suggest that in this medicalised setting, feeding whether it is by tube, bottle or breast, tends to be seen primarily as a productive act of nutrition with less emphasis on relational aspects [4,21,22]. Mothers may feel that they are unable to attend to their baby’s signals and needs and their own needs for closeness and interaction due to an institutionalized environment, which tends to favour a task-oriented approach to infant feeding [4,21]. Studies have shown that mothers want to experience breastfeeding as mutually satisfying and relationship strengthening [4,23] and when feeding comprises of a shared awareness and a balance in emotional and physical needs between the baby and the mother it may be defined as an attuned feeding [23].

With regard to breastfeeding much of the literature relates to the ways in which, in some cultures, women have to negotiate the places and spaces within which they breastfeed [24,25]. This relates, in part, to the sexualisation of women’s breasts in the media making breastfeeding an increasingly private activity to be conducted away from the public gaze [25,26]. Hence, mothers have to negotiate breastfeeding with regard to time, place and space and the presence and approval or disapproval of others. This negotiation of space and place is likely to be particularly challenging within NICUs.

There is a lack of research that explores how place and space influence the relational aspects of infant feeding, particularly from a cross-cultural perspective. Well designed ethnographic research can inform health services in the design and configuration of NICUs to prevent institutionalised parenting, to maximise the quality of parent-infant contact and facilitate breastfeeding. Exploring and describing the impact of space and place on feeding is essential to inform implementation of appropriate interventions to increase parental involvement and facilitate an attuned feeding.

Thus, the aim of this cross-cultural ethnographic study was to explore, in-depth, the impact of place and space on mother’s experiences and practices related to feeding their preterm babies in NICUs in Sweden and England.

## Methods

### Design

An ethnographic approach was utilised to explore a specific aspect of the neonatal environment. Ethnography is an interpretive methodology that provides rich descriptions and attempts to explain the cultural knowledge of a group of people; the research takes place in the field, with people’s actions and accounts being studied in everyday contexts to elicit explicit (more obvious) and tacit (hidden) cultural knowledge [27].

### Setting

In-depth ethnography was conducted in two NICUs in central Sweden and two in the north-west of England. In each country one level 2 and one level 3 NICU were selected, representing variation in levels of intensive care, health care practices related to infant feeding, and use of space and place. At one extreme, in **NICU A (Sweden)**, parents may stay during the intensive care phase in a parental bed next to the incubator in a room shared with up to three other babies/families but with screens in between. After the intensive care phase, for example when ventilation is no longer required, the baby is transferred with the parent(s) to a single room, in which the whole family (including siblings) may stay for the remaing part of the hospital stay. Infants who need less intensive care (e.g. no ventilation) can be transferred with the parent, straight after delivery, to an own room, in which they can stay for the entire hospital stay. **NICU B** (Sweden) and **NICU C** (England) are similar in layout to each other. In NICU B there are beds for parents by the incubators occasionally but not as a standard. There are reclining or comfortable chairs by most incubators or cots in both NICU B and C and in both these NICUs there are designated rooms for parents (5 and 4 respectively) that may either be used as a single room for the family or shared between two mothers. In **NICU D** (England) there are fewer comfortable chairs and there are only four rooms that parents may stay in.

### Participants

Information (oral and written) about the study was presented to the staff before commencing the research at specific staff meetings. If staff agreed to participate a consent form was provided by the first author. Only one member of staff declined to be observed and interviewed. Mothers and fathers were given oral and written information about the study one day or more after the baby was admitted to the NICU. In those cases where the baby was critically ill, information was given when s/he had stabilised. The only criterion for inclusion was that the baby was born preterm (< 37 gestational weeks) and admitted to the neonatal unit. The exclusion criteria were applied to mothers and fathers who experienced temporary or long-term serious medical and mental complications, who did not speak Swedish or English, and who did not wish to participate. Mothers and fathers who agreed to participate signed a consent form which was provided by the first author. Two mothers did not want to participate and one mother withdrew. The recruitment of mothers/fathers was based on strategies of maximum variation and purposeful sampling. The latter was utilised in order to obtain data from mothers/fathers who were followed throughout the hospital stay. Theoretical sampling was utilised, in that participants, parents and staff, were selected in order to inform our developing understanding of the area of investigation.

In total, 52 mothers, 19 fathers and 102 staff were observed and interviewed. A description of the families included is presented in Table 1. In the Swedish population, there were four sets of twins; in the English population there were six sets of twins. The Swedish babies’ mean birth weight was 1735 grams and they stayed 44 days on average. The English babies’ mean birth weight was 1825 grams and they stayed 46 days on average.

**Table 1:** Characteristics of families included (N=52)

	**Swedish A**	**Swedish B**	**English C**	**English D**
Size of NICU (infant’s beds)	20	16	10	29
**Mothers**	n = 9	n = 13	n = 16	n =14
Primiparous	5	5	10	10
Multiparous	4	8	5	4
Age, years	22–45	22–40	19–36	19–36
Single parent	0	1	1	2
Not born in Sweden / England	2	0	1	3
Occupation*				
Managers / Professionals / Tecnicians and associate professionals / Clerks	4	8	4	6
Service and shop workers / Craft workers	1	3	8	3
Elementary occupations	0	0	2	0
Unemployed / housewife	1	2	1	3
Studying	2	0	1	2
Missing	1	0	0	0
Feeding at discharge (breast milk and method)				
Exclusive breast milk, at breast	6	11	4	3
Exclusive breast milk, at breast and bottle	2	1	1	1
Exclusive breast milk, bottle	0	0	1	2
Partial breast milk, at breast and bottle	0	0	3	0
Partial breast milk, bottle	0	0	1	0
No breastmilk	1	1	6	8
**Fathers**	n = 5	n = 7	n = 3	n = 4
Age, years	24–47	28–47	23–40	25–40
Not born in Sweden	0	0	0	1
Occupation*				
Managers / Professionals / Tecnicians and associate professionals / Clerk	1	4	0	0
Service and shop workers / Craft workers / Plant and machine operators	3	1	1	3
Elementary occupations	0	1	1	0
Unemployed	1	1	1	1
Studying	0	0	0	0
Missing	0	0	0	0
**Infants**	n = 12	n = 14	n = 20	n = 16
Female	6	7	15	8
Gestational age at birth, weeks	25–34	29–35	23–35	26–35
Birthweight, grams	550–2015	950–2855	750–2440	745–1960
Length of hospital stay, days	24–114	5–79	7–220	9–95

*Occupations categorised according to the International Standard Classification of Occupations 1988, ISCO-88.

### Data collection

The ethnographic work involved 11 months of participant observation of activities on the NICUs, with particular reference to interactions between NICU staff, mothers, fathers and their babies related to infant feeding. Observations were made during day and night shifts over a period of 3 months in Sweden (May-July 2009), 6 months in England (Sept-Feb 2009–2010) and 2 further months in Sweden (March-April 2010) by the first author who travelled back and forth between the two units. The observations were made by sitting in those rooms where mothers, fathers, and their baby and staff were and were supplemented by interviews with parents and staff that related to what had been observed. During the observations, field notes were taken and where possible interviews were recorded using a digital tape recorder. The observer maintained a ‘moderate’ level of participation [28], i.e. she was not simply a passive observer/spectator but neither was she an active participant who was engaged in staff related activities. In the ‘moderate’ level of participation, a balance is kept between being an insider and an outsider and between participation and observation. In this study, the observer was dressed as any non-staff member would be, positioned in the corner of a room (mostly) and did not participate in care. However, small tasks were sometimes carried out, for example a blanket or a drink was fetched or a pillow was tucked behind the mother’s back. Spradley’s nine-dimension framework for data collection [28] was used as a guide to initial observations. This framework is comprised of nine dimensions that guide observations: the physical space; people involved; related acts people do; objects present; single actions; set of related activities carried out; sequencing over time; goals people try to accomplish; emotions felt and expressed. Later on, more focused observations were made in order to answer the research questions and elicit more specific aspects. In total, 600 hours of fieldwork were performed, of which 300 hours were direct observations and interviews.

Descriptive data on hours and numbers of observations and interviews in Sweden and England are presented in Table 2.

**Table 2:** Observations and interviews conducted with staff, mothers and fathers

	**Sweden**	**England**
**Total (mothers, fathers, staff)**		
Hours of observations	108	102
Hours of interviews	54	42
Number of interviews > 10 min	59	46
Length of interviews	10-120 min (55 min)	10-120 min (55 min)
**Obsevations/interviews separated**		
Number of observations/ interviews with mother	56	20
Number of observations/ interviews with mother and father	20	5
Number of observations/ interviews with parent(s) and staff	39	34
Number of observations/ interviews with staff	25	26

The study was ethically approved in Sweden by The Regional Ethical Review Board, Uppsala and by the National Research Ethics Service and University of Central Lancashire ethics committee in England.

### Analysis

A grounded theory approach was utilised [29,30]. Field notes and interviews with parents and staff were transcribed and data entered into a qualitative software package MaxQDA. The analysis of data involved interpretation of the meanings, functions, and consequences of actions and institutional practices [27]. Transcripts were initially coded to identify concepts in the data; these concepts were then grouped together into preliminary codes. During this phase of the coding, each incident was compared to other already identified concepts through observations and interviews (i.e. constant comparative method) and hence codes. Identified codes and their properties and dimensions constituted a continuously developing ‘framework’ for further observations/interviews. Every incident was coded into as many sub-categories as possible [30]. Theoretical coding was conducted primarily by the first author but continuously discussed and elaborated on with the second author. Codes were collapsed into sub-categories and categories, to develop themes within the data and form linkages and relationships between them, ultimately achieving a level of abstraction and interpretation [29]. Because this was conducted as a comparative ethnographic study, the process moved back and forth between open and theoretical coding. Although the first author ‘fought familiarity’ [27] when doing the field work during the first three months in Sweden, complementary data were needed from the Swedish context after the six months in England to saturate the emergent theory, hence the second data collection period in Sweden. Once all data was collected and preliminary themes and categories were identified, interviews and observations analyzed at the commencement of the data collection period were coded again to enhance the rigor of the analysis. A field-work journal was kept and discussed with the second author. Throughout the open and theoretical coding several global themes emerged. In this paper we only present the data on the impact of space and place on feeding.

## Results

**Figure 1:**
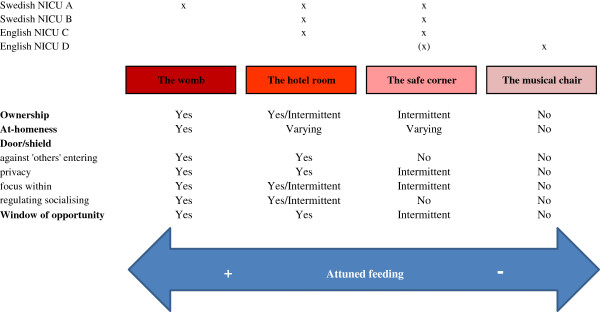
Categories of place and space and their influence on experiences of feeding in relation to four typical places/spaces.

The core category: ‘the room as a conveyance for an attuned feeding’ , comprised four categories and their properties/dimensions of space and place, which strongly influenced the experience of attuned feeding (see Figure 1). Attuned feeding was a concept irrelevant to the method of feeding (i.e. bottle or breast) related to the mother having a sense of connectedness and a shared awareness with her baby, feeling ‘present’ emotionally and tuning in to her baby’s emotional and physical needs. The first of the four categories, ‘**ownership’** of place and space, signalled the mothers’ (and fathers’) importance and role in which the extremes were being ‘a mother’ or ‘a visitor’. Hence, level of ownership related to feelings of being respected and acknowledged as the primary caregiver. Secondly, the level of **‘at-homeness’ ,** in the place, was important as it was accompanied by feelings of warmth, comfort and normality. Thirdly, the place and space functioned as a ‘**door or a shield’**: 1) against ‘others’ entering the space and watching; 2) enabling privacy and close physical contact; 3) facilitating a ‘focus within’ in which mothers’ centre of attention was related to their own baby and those close to the mother and; 4) regulating socialising. Last, the room enabled a **‘window of opportunity’** for feeding in correspondence to the baby’s cues. The categories and their properties of place and space and their influence on experiences of feeding are described below in four typical places/spaces (Figure 1), the ‘womb’ , the ‘hotel room’ , the ‘safe corner’ and the ‘muscial chair’. These rooms represent the place and space where parents were together with their babies. Some parents visited several of the rooms and others only visited one of them.

### The ‘womb’

The ‘womb’ occurred only in NICU A. The ‘womb’ was a place/space where parents and their baby were together as an absolute entity and where no interruption of physical closeness had taken place after birth; the mother, father and their baby had moved to the ‘womb’ straight after birth. If the mother needed intensive care herself, the father brought the baby to the ‘womb’ and as soon as it was possible the mother joined them. There was no negotiation required for the room; parents’ complete ownership of the room was taken for granted, by parents and staff. Hence, parents felt like parents and staff viewed them as parents:

“*Before* [the new unit was built]*, parents couldn’t live here with their babies and then staff had the control. It was breastfeeding that strengthened them in their role. But now, if you get a chance to live with your baby, independent of how you feed, you are close and have control of the situation. We, as staff, see the parents more as parents now.*” (SA11)

There was a tangible feeling of ‘at-homeness’ in the ‘womb’. The room was often quiet and dimly lit with curtains drawn; if a television was on it was on low volume or muted. Parents went to the unit’s kitchen and made meals and then brought the food back to the room. Being in a ‘womb’ did not mean that parents were totally self-sufficient and independent; they were sometimes helped with tube-feeding at nights by staff; they had their baby in a kangaroo position and the staff “*tiptoed*” into the room and tube-fed. The ‘womb’ enabled a focus within, and one mother stated: – “*This room is like a womb. The protective world enables you to just be in it.*” (MA3) To facilitate closeness and minimise medicalisation a wireless monitoring system was used so that the baby could be monitored, by staff, from a station outside the room.

The ‘womb’ also enabled parents to regulate socialising with others. Mothers hardly went outside of the room after birth and tended to have very few visitors even though there were no restrictions on visitors. Thus they did little socialising with other parents and experienced less of the cultural norms and discussions with other parents about their babies.

The ‘window of opportunity’ was wide open in the ‘womb’. Having uninterrupted presence and physical closeness facilitated mothers’ attunement to their babies’ signals. It was a “*flow*” in their communication; as soon as the baby signalled, the mother responded in a way that seemed effortless. During one of the observations, a mother was sitting in her bed alternating between breastfeeding her twins or having them in front of her on the bed, in between her legs. She looked at them most of the time recognizing every movement and never at the clock. When asked about timing of feeding, the mother responded:

“*They are lying with me so I know they get what they need. They relax more when they’re on me, in the sack* [kangaroo wrapping]. *But when they’re like this* [in front of her] *I can see them. Then it’s easier to see their signs of them being hungry. I try not to breastfeed less than every other hour. Sometimes they want to eat every hour. And sometimes, when I have put them down, they start to squirm and then I breastfeed again.. In this room it’s easy to see their signals. I sit and watch them all days. This is how everyone should have it.*” (MA6)

### The ‘hotel room’

The ‘hotel room’ existed in three NICUs (A, B and C). The ‘hotel room’ was a room that mothers themselves asked for or were offered for longer periods. Most mothers were offered a ‘hotel room’ with the baby to share with father, single or to share with another mother, sometime during the stay at the NICU. Large variations were observed in the policies for allowing babies to stay in the parents’ room with regard to baby’s medical state. In NICU A, the wireless monitoring system enabled parents to be with their baby in the ‘hotel room’ as soon as the baby no longer required intensive care. In NICU B and C the baby needed to be more or less without monitors to be with the parents in the ‘hotel room’. Ownership, signalling importance and role of the mother, depended on the availability of the room and duration of occupation. The availability of rooms varied, in NICU A rooms were usually available, in NICU B and C there were fewer rooms and therefore they had to be ‘negotiated’ for. This meant that some mothers were offered a room soon after birth but others later on. Many mothers described the timing for having a room as something that highly affected their sense of being “*a proper carer*”. One mother stated: – “*I could go home and come back but that would have been to visit him and not care for him*.” (MC7) A mother with twins said:

“*I’ve had this room for 2 weeks now. It’s building my confidence up. Having them and getting to know the cues. I have them with me in the room all the time. It’s been invaluable* [the room]*. I think that…when I was up there* [postnatal ward] *I felt distanced from them. I couldn’t be a proper carer. If I’d been able to room in, down here, I think it would have been different.*” (MC3)

The design, facilities and the feeling of ‘at-homeness’ in the ‘hotel rooms’ varied. Some rooms were “*homely and warmer*”, with painted walls, paintings, curtains, cupboards , shower, television, and water boiler, whereas some had less of the ‘at-homeness’ and facilities.

For many mothers, the ‘hotel room’ functioned as a shield to prevent ‘others’ from entering or watching, enabling privacy, described by some as “*enabling a family-life*”. Staff knocked at the door and there were signs to hang on the outside of the room, “*asleep*”, if parents did not want to be disturbed. The staff also recognized the importance of letting mothers “breastfeed and have some peace and quiet without being watched constantly”. One of the mothers described the impact of the ‘hotel room’ as a shield from staff surveillance:

“*In the other room, they* [staff] *watched and commented. Even if they didn’t it felt that way. You have to reach that stage where you feel you’re the mum. And that I know best. And I don’t think you can do that if someone is always around. Right after that we moved to this room I felt I was the mum.*” (MA2)

The ‘hotel room’ facilitated a focus within, as it protected the baby from too many stimuli and disturbances. Mothers who had experienced different rooms could see the difference in their baby’s breastfeeding behavior: – “*In there* [the nursery]*, it’s a bit noisy and people are coming and going. She seems to have a better go if it’s quieter and I am relaxed*.” (MC9) Furthermore, the ‘hotel room’ facilitated mothers in resting and reflecting on everything that had happened and their new role as a mother. One of the mothers described her feelings:

“*We might be here for 8 more weeks. It feels like oceans of time. The parental role is new and you need to find it. I lost my mum when I was 15. So you want to be a good parent. I feel apathetic; there is so much to grip. But I try not to think too much, I try to rest. I watch TV. You can’t cope with your own reality so you try to find one that you can rest in.*” (MA10)

The ‘hotel room’ provided a ‘window of opportunity’; many mothers described that the benefit of having a room was that they were there, they could relax and did not miss their baby’s “*periods of awakeness*”. One mother described how she, the baby and her partner attunded to their baby in this setting:

“*We withdrew from everything. We focused on him and it was peace and quiet and we could hear him. I saw that he was searching so I just put him at the breast and he started to suck and he hadn’t before. It was the breakthrough. There were just a few hours in between feedings. I was enabled freedom. I didn’t look at the clock but I did as he wanted. God how great! We were attuned to him.*” (MB6)

One of the disadvantages of being in one’s own space most of the time and/or for long periods was becoming isolated, a concern of some staff in NICUs A and B who referred to parents becoming “*too isolated*”. This was also expressed by a few mothers, “*sometimes I feel a bit lonely.*” (MA2)

### The ‘safe corner’

The ‘safe corner’ existed in all four NICUs, but to a lesser extent in NICU D. A ‘safe corner’ was described as a private place/space: – “*Here I have my own little corner.*” (MB1) It was evident that it was the mother’s chair/bed and no one else would take it away from her. In NICU A there was always a parental bed next to the incubator. In NICU B, parental beds were more ‘random’. More often, in NICU B and C, there were comfortable chairs placed by the incubators/cots and the bigger chairs were not moved around, they were more ‘static’. In NICU D, although there were rarely chairs by the incubators/cots, some mothers had an ‘assigned’ chair depending on the size of the room and the cot’s location in that room; smaller rooms had more ‘assigned’ chairs and if the cot was located in a corner it seemed as if the chair was more ‘static’. The bed or chair signalled ownership; importance as a parent and what was expected of the parents. A bed signalled that parents were expected to lie in it and that a bed was needed in order to stay, in closeness, with their baby. A chair next to the cot signalled that parents would come and stay close to the baby, possibly holding him/her for as long as they could.

The level of ‘at-homeness’ varied between the NICUs’ safe corners. In NICU A, design, art and colours had been chosen carefully to render a warm but yet ‘clean’ impression. As all families had their own bookshelf next to their bed by the incubator, they had a place to make ‘their own’. NICU B and C had a similar atmosphere in that it was staff who had constructed a homely atmosphere with a ‘personal touch’ (e.g. ornaments, flowery curtains). In NICU D, there was less attention paid to the decoration and ornamentation and the colours on walls and curtains made the environment seem rather ‘cold’ and clinical.

The experience and use of a shield differed. In NICU A there were always screens in place. In NICU B, C and D, there were rarely any screens in place but sometimes placed next to a mother on her request or staff’s inquiry. While some mothers felt they could ask for a screen other mothers did not want to bother the staff. Hence, some mothers felt under surveillance compounding their need to ‘perform’. One mother said: – “*It doesn’t feel natural to breastfeed in this environment. There are so many people around you and many who come up and ask “how’s it going?*” (MB1) By having to ‘perform’ and ‘achieve’ mothers could not fully be attuned to their own and their baby’s emotional and physical needs. A cultural difference was noticeable that related to norms in that country; in Sweden mothers tended to feel more secure about exposing their bodies and breasts. In England the mothers tended to be more concerned and anxious about revealing their breasts: – “*I wouldn’t have breastfed in here. It’s too public and I would never do it in public*.” (MC5) Skin-to-skin contact was very rarely seen in the English units and if so, almost never without a bra on. One English mother was sitting with her son in kangaroo position: – “*There are lots of people walking by. If I had a screen I would have taken it* [bra] *off. In x* [another NICU] *it felt a bit more private ‘cause we were in a corner. And with the screen there I could express by his side.*” (MD4) Hence, both skin-to-skin contact and breastfeeding were hindered by cultural concerns about ‘exposure’ of one’s breasts.

Socialising between mothers was most common in the safe corner, in comparison to the other rooms. Some mothers described the positive aspect of socialising, that they liked having other mothers to socialise with. This included sharing information, supporting each other, “*learning the ropes*”, or talking about football. Some mothers also described negative aspects of socialising, in which the most negative experience was that of comparing oneself negatively to other mothers. In the Swedish NICUs mothers tended to compare their level of being ‘successful’ in breastfeeding with other mothers. This social comparison tended to increase the mothers’ stress and anxiety related to being “*good-enough*”. Furthermore, in the safe corner, parents gained cultural knowledge through the professional discourse and through other parents’ presence and handling of their babies on what to achieve as a parent and how to be with your baby.

Mothers in the ‘safe corner’ had to leave their baby to go to the maternity unit, a room in the unit/hospital or home after visiting. Leaving was troublesome because of a growing sense of attachment. This leaving the ‘window of opportunity’ disrupted the opportunity for closeness and the feeling of being attuned:

“*You don’t get these maternal feelings from the start. It takes time and something that develops. The more closeness she gets, the closer she wants to be. She would feel the best if she was with me constantly but she can’t. She can’t be in my room.*” (MB1)

### The ‘musical chair’

The ‘musical chair’ room existed only in NICU D. Parents and staff described the ‘musical chair’ as a place/space with too few facilities for being present, close or having privacy. In NICU D there were no reclining chairs but wooden or plastic chairs with little comfort. In two of the rooms, the high-dependency room and the nursery, chairs were placed in the centre of the room, around a table, on which the staff had papers and medical records. Within this institutional context the space/place was not negotiated. Thus, the mothers had no ownership of the place and space and were reduced to visitor status. Compared to the other NICUs, parents were not ‘allowed’ to be in the same room as the baby stayed in during medical rounds. Furthermore, in NICU D parents were prohibited to eat or drink anything except water in the same room as their baby was cared for. These ‘policies’ acted as barriers for presence. One mother described the impact of the environment in the musical chair room:

“*If I had had a room I would have stayed here and everything would have been easier. It’s difficult being here ‘cause you don’t have anything to do. If you had had a room you could read a book or watch TV and come in here. But when you don’t, you just sit here. And they’ve told me, before I had her, that I shouldn’t pick her up. That it’s best not to pick her up. So when she’s awake I pick her up and give her a cuddle and then put her back. And I think that’s good ‘cause then they get into a routine.*” (MD9)

Mothers in NICU D were requested to room-in for 24–48 hours prior to discharge, in order for the staff to check how the mother was doing as a mother – that she would manage taking care of her baby. One of the nurses said:

[Pointing at one of the cots] “*She’ll have to room in for a couple of nights ‘because she’s tube-feeding and on oxygen.* [Pointing at another cot] *But that mum won’t have to ‘cause she has other children and there's nothing wrong with him. She can manage.*” (SD35)

In the ‘musical chair’ room there was no feeling of ‘at-homeness’. One of the staff said:

“*This unit feels much busier, obviously it’s a bigger unit, there’s a lot more staff and it feels technical to me. It feels more removed. I think I can imagine how a mum might feel walking in here. I think it might feel a bit colder, not in temperature but because of the unit.*” (SD23)

The lack of comfortable chairs, the bright light and the noise were hindrances for an attuned feeding as mothers could not sit for a long time and their babies’ behavioural development was not acknowledged. Furthermore, there was less socialising between mothers in the ‘musical chair’ room. Mothers did not chat with each other to the same extent as they did in the safe corner. Mothers’ low presence (couple of hours/day), transfer of babies in and out of different rooms and vast space in between cots hindered the development of more secure bonds with other mothers. Mothers tended to mimic other mothers and staff in their behaviour towards babies. As an example, in NICU D, staff tended to bottle feed the babies face-to-face or holding the baby sideways with one hand around the neck and the other hand holding the bottle. Hence, mothers also bottle fed their babies in the same ways and when asked “*why*” they all referred to having seen and been taught by staff. During one of the observations, a mother was feeding her baby sideways:

“*This is what I don’t like. Feeding her. She doesn’t take as much when I feed her. The nurses are better. And I don’t like holding her like this. I didn’t start to. I held her in my arms but then the nurses told me that she was too comfortable and that it made her fall asleep. So they told me to hold her like this but I don’t feel as confident holding her like this. I hold her better like this* [breastfeeding position].” (MD9)

Being in the unit a few hours/day minimized the ‘window of opportunity’ for feeding. One of the nurses said:

“*It’s difficult with breastfeeding ’cause the mums aren’t here that much and they need to be here with their breasts to make it happen. But they’re not. A lot of mums have a lot of social problems. And they have older children at home. And they haven’t established a relational bond. They’ve had their ‘mothering’ interrupted and they don’t feel it’s their baby. They might think they’re in the way and there isn’t really a place for them. They don’t even have comfy chairs. So it’s not easy.*” (SD1)

## Discussion

The aim of this study was to explore, in-depth, the impact of place and space on mothers’ experiences and practices related to infant feeding their preterm babies in NICUs in Sweden and England. Our data revealed that when considering feeding as a relationship, the place and space for this action was of utmost importance with regards to parents’ experiences.

Relationships are central to all human experience and when considering feeding the attention to human interactions must be a priority [22]. Our study shows the importance, from the mother’s and the baby’s perspective, of attunement, in which both the mother’s and the baby’s physical and emotional needs are acknowledged, regardless of method. If disregarding the quality of feeding, it becomes a task with little pleasure for both the mother and the baby [31] which has implications for the developing mother-baby relationship [23]. As feeding is one of the most frequently conducted activities, the impact of the interactional behavior during feeding may influence/steer other interpersonal experiences/interactions [4]. Thus, the way in which NICU is configured needs to take into account what best facilitates an attuned feeding.

Our findings show that when the space and place constructs a separation between mother and baby, it can make the mother feel unimportant, reducing her status to that of a visitor. An ‘ownership of the baby’ as a result of physical closeness, has a fundamental impact on the experience of maternal identity [17,32]. When the place is constructed so that a mother has a sense of ownership of place/space this in turn helps to facilitate her in feeling ownership of her body and baby and supports her in feeling important as a mother and as a person [33,34]. Thus, when space and place is designed so that the mother’s own emotional and physical needs are met and where she can be ‘present’ emotionally it facilitates an attunement in which there is a shared awareness and a balance between the mother and her baby. However, the timing and duration of allocation of place is crucial for the feeling of ownership. In NICU A, the only difference between the ‘womb’ and the ‘hotel room’ was the timing for having an own room, in which mothers in the ‘womb’ had the room straight after birth until discharge. Based on findings from that NICU, as well as from the other NICUs, we would argue that the earlier and the longer a mother can stay with her baby in close proximity, the more she will be attuned to her baby’s signals and be ‘in charge’ of her baby’s care.

To our knowledge, the feeling ‘at-homeness’ has previously not been acclaimed to be of importance in relation to neonatal care. However, the phenomenon of ‘at-homeness’ has been studied in other care settings where patients are admitted for a prolonged time, such as in dementia and palliative care. Three concepts of atmospheres (i.e. ‘at-homeness’) have been identified: hospitality, safety and ‘everydayness’ [35,36]. In our study, observations showed that the atmosphere of hospitality was evident in the ‘at-homeness’ as people felt welcomed and/or experienced material things that made the environment a bit better. A warm atmosphere was signalled when attention was paid to the interior design as it conveyed ideas that the care given was warm and caring. In contrast, a sterile environment conveyed ideas that the care was cold and sterile. The third concept, ‘everydayness’, was the most evident aspect of at-homeness in our study as it depicted a de-institutionalization in which the environment became more home-like (e.g. a pleasant view from the window or paintings). Hence, we argue that when a sense of at-homeness is evident in the NICU, it provides a *spatial* “safe holding environment” [37], in which the healing conditions become more optimal.

Numerous studies show the importance of ‘privacy’, in neonatal care [13] as well as during breastfeeding [24,38]. However, the need for parental privacy may be in tension with percieved health professional imperatives to maintain surveillance of the patient, in this case the baby described by Johnson et al. [39], as an aspect of professional power. Thus, the provision of new medical technology and medical staffs’ needs for observation and supervision are often accommodated in the NICUs, whereas the parents’ and infants’ needs are less well addressed [40]. White [41] has argued that future designs of NICUs should be planned to facilitate as much proximity as possible by changing the locus of care from the incubator/cot to the parents’ arms. Such a change will have beneficial effects not only for breastfeeding [42,43] but also on the baby’s neurological development, the parental sense of confidence and trust and the parent-baby relationship [5,6].

It has been suggested that parents may feel isolated if they have less opportunities to interact with others [5,44] although, to the contrary, it has been suggested that parents do not necessarily want to be exposed to other parents and need to be alone [34]. It was clear in our study that the configuration of space influenced the opportunities for and nature of socialising. Parents did not express feelings of being isolated when they had never spent time in communal rooms in NICUs (i.e. parents who experienced the ‘womb’ in NICU A), only when they had experienced both. Our findings indicate that what is important is to facilitate parental opportunities to regulate when and with whom they want to socialize, through the designs of NICUs.

### Strengths and limitations

To our knowledge, this is the first cross-national ethnographic study conducted in NICUs. In addition, it is one of the few hospital based, cross-national ethnographic studies; it involved almost a year of field work, in four NICUs, in two countries. Usually ethnography is only conducted in one or more local settings but in this study we included very different NICUs in terms of spatiality and ‘breastfeeding culture’. However, as we sought maximum variation in NICUs, within and between countries, we chose a design where the first author went back and forth between the NICUs in each country to, at least, attempt to represent a temporal variation of the parents studied. Hence, some parents were followed from the infant’s birth throughout the hospital stay.

This research was conducted in an overt manner, in which the first author used a moderate level of participation [28]; this may have influenced the behaviour of the people who are studied, however, over time this effect appeared to lessen as they habituated to the presence of the researcher [27].

A potential limitation of the study is that the first author had worked in neonatal care for more than 10 years and was a native Swede bringing some familiarity and preconceptions [27]. In order to enhance credibility, i.e. whether or not the research findings represent a credible conceptual interpretation of the data and thereby trustworthiness [45], field notes and the field-work diary were discussed with the second author who is English and less immersed in neonatal care. Furthermore, a third data collection period was added to the original design in order to further explore issues that had arisen.

## Conclusion

This study illustrates the importance of spatial configuration of NICUs on parental experiences, parent-infant attunement, infant feeding practices and ways and degrees of socialising with other parents. Given the impact of this it seems crucial that NICU design and reconfigurations take considerable account of the types of setting that maximise parent-infant contact and closeness. The configuration of place and space also appears to influence the extent to which an environment is experienced as institutionalised by parents and staff.

## Competing interests

The authors declare that they have no competing interest in the present research.

## Authors’ contribution

RF and FD designed the study. RF conducted the field work including observations and interviews. RF conducted the analysis and FD participated in the ongoing discussions of the data and analysis. Both authors drafted and wrote the manuscript and approved the final version.

## Author’s information

RF works as a Senior Lecturer at the School of Health and Social Studies, Dalarna University, Sweden and since 2012 as a Senior Research Fellow at the Maternal and Infant Nutrition and Nurture Unit (MAINN), School of Health, University of Central Lancashire, UK. FD is a Professor in Maternal and Infant Health and the Director of the Maternal and Infant Nutrition and Nurture Unit (MAINN), School of Health, University of Central Lancashire, UK ; she is also Visiting Professor at the School of Health and Social Studies, Dalarna University, Sweden and Adjunct Professor at University of Western Sydney.

## Pre-publication history

The pre-publication history for this paper can be accessed here:

http://www.biomedcentral.com/1471-2393/13/179/prepub
